# Understanding Drivers of Vaccine Hesitancy During the COVID-19 Pandemic Among Older Adults in Jiangsu Province, China: Cross-sectional Survey

**DOI:** 10.2196/39994

**Published:** 2023-02-07

**Authors:** Liuqing Yang, Lili Ji, Qiang Wang, Guoping Yang, Shixin Xiu, Tingting Cui, Naiyang Shi, Lin Zhu, Xuepeng Xu, Hui Jin, Shiqi Zhen

**Affiliations:** 1 Department of Epidemiology and Health Statistics School of Public Health Southeast University Nanjing China; 2 Key Laboratory of Environmental Medicine Engineering, Ministry of Education School of Public Health Southeast University Nanjing China; 3 Jiangsu Provincial Center for Disease Control and Prevention Nanjing China; 4 Department of Immunization Planning Wuxi Center for Disease Control and Prevention Wuxi China

**Keywords:** vaccine hesitancy, older adults, socioeconomic status, vaccination history, COVID-19, 3Cs model, confidence, complacency, and convenience

## Abstract

**Background:**

Older adults are particularly at risk from infectious diseases, including serve complications, hospitalization, and death.

**Objective:**

This study aimed to explore the drivers of vaccine hesitancy among older adults based on the “3Cs” (confidence, complacency, and convenience) framework, where socioeconomic status and vaccination history played the role of moderators.

**Methods:**

A cross-sectional questionnaire survey was conducted in Jiangsu Province, China, between June 1 and July 20, 2021. Older adults (aged ≥60 years) were recruited using a stratified sampling method. Vaccine hesitancy was influenced by the 3Cs in the model. Socioeconomic status and vaccination history processed through the item parceling method were used to moderate associations between the 3Cs and hesitancy. Hierarchical regression analyses and structural equation modeling were used to test the validity of the new framework. We performed 5000 trials of bootstrapping to calculate the 95% CI of the pathway’s coefficients.

**Results:**

A total of 1341 older adults participated. The mean age was 71.3 (SD 5.4) years, and 44.7% (599/1341) of participants were men. Confidence (b=0.967; 95% CI 0.759-1.201; *P*=.002), convenience (b=0.458; 95% CI 0.333-0.590; *P*=.002), and less complacency (b=0.301; 95% CI 0.187-0.408; *P*=.002) were positively associated with less vaccine hesitancy. Socioeconomic status weakened the positive effect of low complacency (b=–0.065; *P*=.03) on low vaccine hesitancy. COVID-19 vaccination history negatively moderated the positive association between confidence (b=–0.071; *P*=.02) and lower vaccine hesitancy.

**Conclusions:**

Our study identified that confidence was the more influential dimension in reducing vaccine hesitancy among older adults. COVID-19 vaccination history, as well as confidence, had a positive association with less vaccine hesitancy and could weaken the role of confidence in vaccine hesitancy. Socioeconomic status had a substitution relationship with less complacency, which suggested a competitive positive association between them on less vaccine hesitancy.

## Introduction

Older adults are at risk of illness, serious complications, hospitalization, and death from vaccine-preventable diseases owing to declining immunity associated with aging [1,2]. Vaccine-preventable diseases, which are common in older adults, such as influenza, pneumococcal disease, and shingles, pose a considerable disease burden worldwide [3-5]. During the COVID-19 pandemic, patients with respiratory viral infections are vulnerable to subsequent bacterial infections, and coinfections lead to increased disease severity and mortality [6]. Most countries have included older adults as a priority group for immunization programs. However, even in high-income countries, older adults’ vaccination coverage remains below the World Health Organization’s targets [7-9].

“Vaccine hesitancy,” proposed by the Strategic Advisory Group of Immunologists working group, could considerably hinder vaccinations. This term was defined as “delayed acceptance or refusal of vaccines despite their availability” [10]. Several studies have demonstrated that theory-based behavioral interventions could be more effective than other interventions [11-14]. Hence, various theoretical frameworks have been proposed to solve vaccine hesitancy and address the complexity of vaccine hesitancy measurement. The “3Cs” (confidence, complacency, and convenience) model, which is a professionally validated theoretical framework for vaccine hesitancy, is also considered one of the most useful models of vaccine hesitancy because it is concise, intuitive, and easy to understand and apply [10,15]. The 3Cs model demonstrates the influence of psychosocial factors, which are thought to provide the best explanation for deferring or deciding to vaccinate [16,17].

The causes of vaccine hesitancy among older adults were summarized by the following factors, including misinformation, perception of good health, perception of vaccine ineffectiveness, side effects, and distrust of the health care system [18]. Many studies without a theoretical framework focused on exploring more factors that were associated with older adults’ vaccine intention potentially [19,20]. Moreover, studies that used health theoretical frameworks, including the 3Cs model, may focus on quantitative studies, where demographic characteristics may not be the priority research variables [21]. Sociodemographic characteristics and context have been confirmed by many studies as important influencing factors for vaccine hesitancy [22,23]. Moreover, vaccination history, which strongly impacted vaccination intentions in earlier studies [24,25], requires exploration in current frameworks.

As China is the most populous country in the world, the number of adults aged ≥60 years (267 million in 2022) is larger than in any other country [26]. However, during the COVID-19 pandemic, approximately 35.83 million older adults have not completed the full vaccination dosage, of which 24.6 million have not received at least one dose of the vaccine [27]. This study aimed to develop a model to measure vaccine hesitancy among older adults based on the 3Cs framework with socioeconomic status (SES) and vaccination history as moderators. Further, an attempt was made to distinguish the association and degree of influence between these factors, thus providing evidence for precise interventions to improve vaccination rates in older adults.

## Methods

### Participants and Procedure

A cross-sectional questionnaire survey was conducted between June 1 and July 20, 2021, using a stratified sampling method. We assessed vaccine hesitancy among adults aged ≥60 years. After stratifying by economic level, the cities of Changzhou and Yancheng were selected as the survey areas for this study, among 13 cities in Jiangsu province, China, using the random number method. The gross domestic product per capita in Jiangsu province in 2019 (US $17,918) was used as the cutoff between high (Changzhou: US $22,670) and low (Yancheng: US $11,473) economic levels [28]. Then, with the county/district as the smallest selection unit, Wujin district and Liyang county in Changzhou and Yandu district and Dongtai county in Yancheng were sampled as the locations for this survey, using the random number method.

Older adults who visited designated medical checkup clinics for annual physical examinations, which were government-organized checkups for all eligible residents within that residence, were recruited as participants. Participation was voluntary. All adults aged older than 60 years were encouraged to participate. The one-on-one interview survey method was adopted, and all investigators were trained uniformly. Those with cognitive impairment were excluded, as were those who had a relative complete the survey for them. The calculation of the sample size required for this study was shown in Material S1 in Multimedia Appendix 1. The inclusion and exclusion criteria of the participants in this study were presented in Figure S1 in Multimedia Appendix 1.

### The Measurement Items and Hypotheses of the Questionnaire

The questionnaire consisted of 3 sections. The first section was demographic (age, sex, marital status, and self-assessment of financial situation), SES, and health information. Individual SES was evaluated by survey area (low or high economic level), education (primary or lower or secondary school or higher), medical career background (yes or no), occupation (retired, government agency or service, or production industry), and monthly revenue (based on the categories of the questionnaire [28]: ≥US $435 or <US $435) [29-31]. The health information included chronic disease history, self-assessment of health status, and vaccination history. Vaccination history consisted of four items: (1) “Have you ever received the herpes zoster vaccine?” (2) “Have you ever received the COVID-19 vaccine?” (3) “Have you received an influenza vaccine in the past year?” and (4) “Have you ever received the pneumonia vaccine?”

The second section was the construction of the 3Cs model, which included confidence, convenience, and complacency dimensions. All items within each construct were chosen according to MacDonald’s view on the definition of the 3Cs model [10], which was presented in Material S2 in Multimedia Appendix 1. The scale of confidence included 2 parts: confidence in the vaccine and confidence in health care workers and vaccine manufacturers [10]. The first part included 4 items [32]: “I think the vaccine is safe/effective/ important” and “I believe that the full chain of vaccine management is safe and effective.” The second part included 4 items: (1) “I trust doctors and nurses”; (2) “I trust hospitals and community vaccination clinics”; (3) “I trust vaccine manufacturers”; and (4) “I trust the vaccine information provided by the government.”

We measured convenience using four questionnaire items: (1) “The poor quality of service at the vaccine clinic would make me not want to go for vaccination”; (2) “It was easy and took me a short time to get the vaccination”; (3) “I can get the vaccine that I want”; and (4) “I can afford the vaccine.” Complacency was measured using four items: (1) “If I do not get vaccinated, I will not get the disease”; (2) “Natural immunity is better than that produced by vaccination”; (3) “The probability of getting a disease is low, so I do not need to get vaccinated”; and (4) “Even if I get infected with a disease I can resist it, so I do not need to be vaccinated.” For structural consistency, we shifted all items in the complacency dimension.

In addition, a vaccine hesitancy scale was included in the third section, which included three items: (1) “How likely would you go for a COVID-19 vaccine?” (2) “Would you get an inﬂuenza shot this year?” and (3) “If you could now get an influenza vaccine at your own expense, what is your choice?” Each item in the second and third sections was assessed using a 5-point Likert scale. A higher score chosen for each item in the second section means higher confidence, a greater level of complacency, and a higher level of convenience in the vaccine. A higher score chosen for the third section (vaccine hesitancy) means less vaccine hesitancy. The detailed information and the original questionnaire are presented in Material S2 in Multimedia Appendix 1.

We proposed 5 hypotheses according to our purpose:

- Hypothesis 1: More confidence is positively associated with less vaccine hesitancy.
- Hypothesis 2: More convenience is positively associated with less vaccine hesitancy.
- Hypothesis 3: Less complacency is positively associated with less vaccine hesitancy. (Scores on the complacency dimension would be shifted in the following analysis, ie, a higher score means a lower level of complacency).
- Hypothesis 4: Vaccination history moderates the strength of the association between confidence (Hypothesis 4a), convenience (Hypothesis 4b), and complacency (Hypothesis 4c) with less vaccine hesitancy.
- Hypothesis 5: SES moderates the strength of the association between confidence (Hypothesis 5a), convenience (Hypothesis 5b), and complacency (Hypothesis 5c) with less vaccine hesitancy.

### Measurement Model Testing and Fitting

A confirmatory factor analysis was conducted to assess the reliability and validity of each scale. Reliability was evaluated by calculating squared multiple correlations and composite reliability [33,34]. This study also examined parameter estimates and *t* values, as well as factor loadings and average variance extracted (AVE) [35]. Validity was determined by calculating the square root of the AVE for each latent variable.

According to Gerbing et al [36], the goodness-of-fit index (GFI), adjusted goodness-of-fit index (AGFI), comparative fitness index (CFI), and the root means square error of approximation (RMSEA) were used to evaluate the model fit. GFI, CFI, and AGFI greater than 0.90 indicate a relatively good data fit; RMSEA less than 0.08 indicates good model-data fit [36,37].

### Statistical Analysis

To minimize bias from the exclusion of any missing data for the older adults, we used multivariate multiple imputations to fill in missing values. The *MICE* package in R software (R Foundation for Statistical Computing) was used to complete this imputation. Sensitivity analyses were also conducted for the original data and where the missing data were deleted. Participants’ characteristics were described with mean (SD; for continuous variables) and frequencies (percentages; for categorical and ordinal variables). The variables of SES and vaccination history were processed using the item parceling method [38]. All items related to SES were converted to a 2-category variable (0 or 1) as the cutline described in “the measurement items and hypotheses of the questionnaire” section. Then, the assignment of the items related to the SES and vaccine history dimension were summed, transformed into continuous variables, and standardized [38]. Hierarchical moderator regression analyses were used to examine the moderating effects, and all variables were standardized [39]. Control variables, including demographic and health information, were entered as block 1, followed by the main effects in block 2, which were reflected by the 3Cs dimensions, SES, and vaccination history. Finally, the moderating effects between SES and vaccination history with confidence, convenience, and complacency were used as block 3. Specifically, the regression equation was analyzed in 3 hierarchical steps, and the association between all 3 blocks and the dimension of vaccine hesitancy were explored (Material S3 in Multimedia Appendix 1). Given the impact of the COVID-19 pandemic, COVID-19 vaccination history was performed in the model separately. Structural equation modeling was performed with AMOS software (Statistics Solutions) to test the hypothesized model and estimate the structural coefficients between scales. Bootstrapping (5000 trials) was used to calculate the 95% CI of coefficients [40]. Hierarchical moderator regression analyses were performed using SPSS software (version 21.0, IBM Corp). Statistical significance was set at *P*<.05.

### Ethics Approval

Before completing the questionnaire, it was mandatory for all participants to provide verbal informed consent. They were informed that this was an anonymous survey and that all data would remain confidential. Participants would receive US $2 worth of gifts in return for completing the survey. The Ethics Committee of the Wuxi Center for Disease Control approved this study (2020No10).

## Results

### Participants’ Characteristics

Initially, 1384 participants (response rate: 1384/1591, 87%) took part in this survey. Based on the inclusion and exclusion criteria, a total of 1341 participants were included finally (Table 1). Of these, 28.7% (n=385) had a secondary school education or higher. The mean age was 71.3 (SD 5.4), and 44.7% (n=599) were men. Moreover, 18.6% (n=249) of the participants were single (unmarried, divorced, or widowed). The percentage of participants with health care–related work experience was 1.4% (n=19). The proportions of monthly income less than US $145 and the range of US $145-435 were the highest at 50.9% (n=683) and 31.2% (n=419), respectively. The proportion of participants who assessed themselves as having at least an adequate financial situation was 64.6% (n=867). The most frequently reported self-assessment of health status was “well,” accounting for 45.1% (n=605), followed by “average” (n=434, 32.4%) and “poor” (n=240, 17.9%); further, the proportions of “very well” and “very poor” were 4.1% (n=55) and 0.5% (n=7), respectively. Detailed information is presented in Table 1.

Of the specific vaccines, the percentages of older adults who self-reported having received herpes zoster vaccine, influenza vaccine (last year), and pneumonia vaccine were 0.8% (n=11), 1.5% (n=20), and 0.1% (n=2), respectively. A total of 30.9% (n=415) of the participants had received at least one dose of the COVID-19 vaccine. Additionally, 21.4% (n=287), 3.7% (n=50), and 29.9% (n=401) of the participants self-reported choosing uncertainty, postponing, and refusing vaccination, respectively. Further, 45% (n=603) of the participants were willing to receive vaccines.

**Table 1:** Participants’ primary sociodemographic information.

Sociodemographic information			Participant (N=1341), n (%)
**Sex**			
	Male	599 (44.7)	
	Female	742 (55.3)	
**Marital status**			
	Married	1092 (81.4)	
	Unmarried	18 (1.3)	
	Divorced	8 (0.6)	
	Widowed	223 (16.6)	
**Education**			
	Illiterate or semiliterate	484 (36.1)	
	Primary school	472 (35.2)	
	Secondary school	267 (19.9)	
	High school	97 (7.2)	
	College or equivalent or above	21 (1.6)	
**Medical career background**			
	Yes	19 (1.4)	
	No	1322 (98.6)	
**Occupation**			
	Retirement	370 (27.6)	
	Government agencies and institutions	9 (0.7)	
	Enterprises, commerce, and service industry	32 (2.4)	
	Agriculture, forestry, animal husbandry, fishery, and water conservancy production personnel	610 (45.5)	
	Military	2 (0.1)	
	None	266 (19.8)	
	Others	52 (3.9)	
**Income** **(US $/month)**			
	<145	683 (50.9)	
	145 to <435	419 (31.2)	
	435 to <725	173 (12.9)	
	725 to <1450	57 (4.3)	
	1450 to <2900	8 (0.6)	
	≥2900	1 (0.1)	
**Self-assessment of financial situation**			
	Very generous	10 (0.7)	
	Generous	131 (9.8)	
	Roughly adequate	726 (54.1)	
	Tough	422 (31.5)	
	Very tough	52 (3.9)	
**Self-assessment of health status**			
	Very poor	7 (0.5)	
	Poor	240 (17.9)	
	Average	434 (32.4)	
	Well	605 (45.1)	
	Very well	55 (4.1)	
**Chronic diseases**			
	Yes	774 (57.4)	
	No	567 (42.3)	
**Herpes zoster vaccination history**			
	Yes	11 (0.8)	
	No	1330 (99.2)	
**COVID-19 vaccination history**			
	Yes	415 (30.9)	
	No	926 (69.1)	
**Influenza vaccination history (last year)**			
	Yes	20 (1.5)	
	No	1321 (98.5)	
**Pneumonia vaccination history**			
	Yes	2 (0.1)	
	No	1339 (99.9)	

### Measurement Model Testing and Fitting

The items of each scale were censored based on the factor loadings, followed by the modification indices. Items with factor loadings below 0.5 were not retained, principally. The modified questionnaire is presented in Table S1 in Multimedia Appendix 1. Composite reliability values were greater than 0.6 for each scale (ie, 0.818 for complacency, 0.740 for convenience, 0.786 for confidence in the vaccine, 0.814 for confidence in health care workers and vaccine manufacturers, and 0.732 for vaccine hesitancy). All data are presented in Table S2 in Multimedia Appendix 1. Although the square root of the AVE of convenience (0.651) was slightly lower than the related correlation with complacency (0.680), the square root of the AVE of all other dimensions exceeded the related correlations (Table S3 in Multimedia Appendix 1). The items were also reliable and valid when evaluated based on each item’s error variance and residual covariation, which are presented in Tables S4 and S5 in Multimedia Appendix 1. Additionally, the overall model achieved a good fit with GFI=0.938, AGFI=0.916, CFI=0.929, and RMSEA=0.064. Therefore, the measurement and structural model were acceptable. 

### Hierarchical Moderator Regression Analysis

Married participants were more willing to be vaccinated than their counterparts (*P*=.003), whereas single participants (unmarried, divorced, or widowed) were more hesitant. Age, sex, chronic diseases, self-assessment of health status, and self-assessment of the financial situation were not associated with vaccine hesitancy (all *P*>.05).

Compared with the model in block 1, where control variables were presented, significant changes were made in block 2 (∆*R*^2^=0.156; *P*<.001). Of these, confidence (b=0.244; *P*<.001), convenience (b=0.233; *P*<.001), SES (b=0.150; *P*<.001), and vaccination history (b=0.144; *P*<.001) were all significantly and positively associated with less vaccine hesitancy. Complacency was not significantly associated with vaccine hesitancy (*P*=.82). In block 3, the added moderators, SES and vaccination history, also had significant changes, compared with block 2 (∆*R*^2^=0.017; *P*<.001). SES moderated the impact between confidence (b=0.083; *P*=.002) and complacency (b=–0.065; *P*=.03) with vaccine hesitancy. Convenience (b=–0.057; *P*=.06) was not statistically significant. Therefore, SES can positively moderate the positive effects of confidence on reducing vaccine hesitancy. In other words, the higher SES in people with higher confidence scores was associated with less vaccine hesitancy. SES negatively moderated the positive effect of less complacency on less vaccine hesitancy, which meant that lower SES weakened this positive effect between complacency and vaccine hesitancy. In addition, SES had a substitution effect with complacency. The positive effect of less complacency on vaccine acceptance was more pronounced when the degree of SES was low; however, as the degree of SES increased, the positive effect of less complacency decreased.

Vaccination history had no moderating effect on the association between confidence (*P*=.06), less complacency (*P*=.14), and convenience (*P*=.16) with lower vaccine hesitancy. Hence, hypotheses 4a, 4b, 4c, and 5b were rejected, and hypotheses 5a and 5c were accepted. All detailed information about hierarchical moderator regression analysis is presented in Tables 2 and 3. Related results that were found on the original data where the missing values were not imputed and the data where the missing values were deleted were all presented in Tables S6-S9 in Multimedia Appendix 1.

In the COVID-19 vaccination history subgroup (Table 4), the moderating effect of vaccination history was different. Particularly, the positive association between confidence and less vaccine hesitancy was negatively moderated by COVID-19 vaccination history (b=–0.071; *P*=.02). COVID-19 vaccination history had a substitution effect with confidence. The positive effect of COVID-19 vaccination history on less vaccine hesitancy was more pronounced when the degree of confidence was low. Complacency and convenience were not moderated by COVID-19 vaccination history. In the herpes zoster, influenza, and pneumonia vaccination history subgroup (Table 4), vaccination history was not a moderator between the 3Cs dimensions and less vaccine hesitancy—the same with the overall model.

**Table 2:** Model information in hierarchical moderator regression analysis.

Model	Statistics estimate				Statistics change		
	*R* ^2^	adjusted *R*^2^	SE	∆*R*^2^		*F* test (*df*)	*P* value
Block 1^a^	0.054	0.045	0.977	0.054		6.311 (1328)	<.001
Block 2^b^	0.210	0.200	0.894	0.126		52.438 (1323)	<.001
Block 3^c^	0.228	0.214	0.887	0.017		4.879 (1317)	<.001

^a^The variables in block 1 were age, marital status, gender, self-assessment of the financial situation, self-assessment of health status, and chronic diseases.

^b^The variables in block 2 was age, marital status, gender, self-assessment of the financial situation, self-assessment of health status, chronic diseases, standardized of confidence, convenience, and complacency, and standardized of socioeconomic status and vaccine history.

^c^The variables in block 3 was age, marital status, gender, self-assessment of the financial situation, self-assessment of health status, chronic diseases, standardized of confidence, convenience, and complacency, standardized of socioeconomic status and vaccine history, socioeconomic status*convenience, socioeconomic status*confidence, socioeconomic status*complacency, vaccine history*convenience, vaccine history*confidence, and vaccine history*complacency.

**Table 3:** Variable coefficients of block 3 in hierarchical moderator regression analysis.

Variables		Unstandardized estimate, b (SE)	Standardized estimate	*t* test (*df*=1317)	*P* value
Constant		–0.049 (0.565)	N/A^a^	–0.087	.93
Age		–0.007 (0.005)	–0.039	–1.485	.14
Gender^b^		–0.049 (0.051)	–0.024	–0.960	.34
Marital status^c^		–0.195 (0.065)	–0.076	–2.988	.003
Chronic diseases^d^		0.014 (0.052)	0.007	0.273	.79
**Self-assessment of the financial situation (“very generous” as reference)**					
	Generous	0.100 (0.295)	0.030	0.340	.73
	Roughly adequate	0.054 (0.290)	0.027	0.187	.85
	Tough	–0.028 (0.293)	–0.013	–0.094	.93
	Very tough	–0.023 (0.317)	–0.004	0.072	.94
**Self-assessment of health status (“very poor” as reference)**					
	Poor	0.615 (0.345)	0.236	1.784	.08
	Average	0.662 (0.343)	0.310	1.927	.05
	Well	0.564 (0.343)	0.281	1.644	.10
	Very well	0.384 (0.362)	0.076	1.060	.29
Socioeconomic status		0.132 (0.032)	0.132	4.096	<.001
Vaccination history		0.137 (0.028)	0.137	4.876	<.001
Confidence		0.219 (0.027)	0.219	8.144	<.001
Complacency		0.008 (0.030)	0.008	0.271	.79
Convenience		0.265 (0.034)	0.265	7.870	<.001
Socioeconomic status*confidence		0.083 (0.027)	0.081	3.074	.002
Socioeconomic status*convenience		–0.057 (0.030)	–0.061	–1.913	.06
Socioeconomic status*complacency		–0.065 (0.030)	–0.066	–2.183	.03
Vaccination history*confidence		–0.055 (0.028)	–0.053	–1.923	.06
Vaccination history*convenience		0.045 (0.032)	0.046	1.403	.16
Vaccination history*complacency		–0.047 (0.032)	–0.047	–1.474	.14

^a^N/A: not applicable.

^b^Gender is a binary variable and used “male” as reference.

^c^Marital status was changed into a binary variable, of which unmarried, divorced, and widowed were combined into “single.”

^d^Chronic disease is a binary variable and used “yes” as reference.

**Table 4:** The moderating effect in the vaccination history subgroups.

Variables		Unstandardized estimate, b (SE)	Standardized estimate	*t* test (*df*=1317)	*P* value
**COVID-19 vaccination history subgroup**					
	Vaccination history*confidence	–0.071 (0.030)	–0.067	–2.381	.02
	Vaccination history*convenience	0.033 (0.033)	0.031	0.980	.33
	Vaccination history*complacency	–0.008 (0.033)	–0.008	–0.248	.80
**Herpes zoster, influenza, and pneumonia vaccination history subgroup**					
	Vaccination history*confidence	–0.012 (0.027)	–0.012	–0.445	.66
	Vaccination history*convenience	0.053 (0.032)	0.073	1.671	.10
	Vaccination history*complacency	–0.071 (0.038)	–0.092	–1.882	.06

### Structural Equation Model of Vaccine Hesitancy

To avoid the effects of multicollinearity, we calculated the effects of each of the 3 dimensions of confidence, convenience, and complacency on older adults’ vaccine hesitancy separately. Then, a full model was constructed, where the effects were split equally to solve multicollinearity [41,42]. As with the results from the hierarchical regression analyses, bootstrapping (5000 trials) showed that confidence (b_direct effect_=0.967; *P*=.002) and convenience (b_direct effect_=0.458; *P*=.002) exhibited a significant positive association with less vaccine hesitancy, thus confirming hypotheses 1 and 2. Unlike the hierarchical regression analysis, in the structural equation model, less complacency had a statistically significant effect on less vaccine hesitancy (b_direct effect_=0.301; *P*=.002). Since the structural equation model considered the potential of multicollinearity, whereas the regression analysis did not, the result in the complacency dimension is the more reliable one. Detailed information is shown in Table 5. Sensitivity analysis results conducted on another 2 data sets were all presented in Tables S10-S11 in Multimedia Appendix 1.

The confidence dimension was assigned 2 components: participants’ confidence in the vaccines, and participants’ confidence in the manufacturer who provided the vaccine and in the health care workers who administered it to them. The results for the second-order constructs showed that the direct effects (unstandardized estimate) between confidence in the vaccine confidence and confidence in health care workers and vaccine manufacturers confidence were 1.000 and 0.810, respectively.

**Table 5:** Effects on structural equation model.

Direct effects		Point estimate		Bootstrapping (5000 times)		
		Standardized estimate (SE)	Unstandardized estimate	Percentile, 95% CI	Bias-corrected percentile, 95% CI	*P* value
**Model 1**						
	Confidence–vaccine hesitancy	0.451 (0.113)	0.967	0.758-1.200	0.759-1.201	.002
**Model 2**						
	Complacency–vaccine hesitancy	0.202 (0.055)	0.301	0.188-0.410	0.187-0.408	.002
**Model 3**						
	Convenience–vaccine hesitancy	0.324 (0.063)	0.458	0.331-0.589	0.333-0.590	.002
**Full model**						
	The effects of confidence, convenience, and complacency were split equally	0.112 (0.025)	0.217	0.166-0.265	0.166-0.266	.002

## Discussion

### Principal Results

In this study, a 3Cs model moderated by SES and vaccination history was developed in the group of older adults. The explanation power of the new framework was examined, and the findings supported that confidence, complacency, and convenience all played an important role in older adults’ vaccine hesitancy. Additionally, SES was a significant moderator of confidence and complacency that scales with vaccine hesitancy. COVID-19 vaccination history moderated the association between confidence and vaccine hesitancy. The dual testing of the hierarchical moderator regression and structural equation modeling analyses made the results more reliable and robust.

### Comparison With Prior Work

Analysis of the 3Cs model revealed several factors that influenced older adults' vaccination perceptions. Our findings in the confidence dimension among Chinese older adults were consistent with studies conducted in other countries, such as Peru [43,44], European countries [45], the United States [45,46], and Japan [47]. Skepticism about vaccines and distrust of physicians and the vaccine system contributed to the occurrence of vaccine hesitancy [48]. Moreover, our findings identified complacency that reduced older adults’ willingness to be vaccinated, particularly the preference for natural immunity and misconceptions about the probability and severity of disease occurrence. In contrast to studies in Western countries where older adults were more likely to be vaccinated because of fear of infectious diseases [49-51], older adults in China appeared to be less aware of the risks of infectious diseases. The cultural context of traditional Chinese medicine may provide an explanation for this unique finding, with older adults believing that a healthy body and autoimmunity were the best ways to protect themselves, rather than the pharmaceutical interventions of Western medicine [52]. Vaccination access, including transportation, cost, and vaccine accessibility, also had a high effect on vaccine hesitancy, which differed from other adults or vulnerable groups (eg, pregnant women [53] and children [54]).

The positive moderating effect between SES and the confidence dimension suggested that older adults with high SES had less vaccine hesitancy owing to better educational attainment and higher income than their counterparts. The positive effect of lower complacency on reducing vaccine hesitancy was more pronounced at lower SES. In other words, as SES increased, the positive effect of low complacency gradually decreased. This suggested a substitution effect between low complacency and SES on vaccine hesitancy among older adults. For disadvantaged older adults, complacency was exacerbated by low SES because of low knowledge level and social media occlusion [55]. In the COVID-19 pandemic, low SES was strongly associated with higher COVID-19 cases and mortality [56]. To the best of our knowledge, few studies have explored SES as a moderating variable for its association with vaccine hesitancy among older adults. Many studies explored the association between individual demographic characteristics, such as lower education level and wealth value, with less vaccination intention [57,58]. Hence, this study was more systematic and comprehensive in considering the association with vaccine hesitancy among older adults by considering SES as a holistic indicator.

The difference in the moderating effect of COVID-19 vaccination history and the other vaccines indicated that the promotion of getting COVID-19 vaccination was a success among the older adults in China. The findings presented the substitution relationship between COVID-19 vaccination history and confidence, which was not presented in the other vaccines history group. Although vaccination history (either COVID-19 or other) did not reduce older adults’ complacency, their hesitancy would be decreased after COVID-19 vaccination by influencing confidence, which was a more powerful indicator of vaccine hesitancy in this study.

Marital status was the significant demographic factor that could influence vaccine hesitancy among older adults. As summarized in a large review, positive perceptions and encouragement of vaccination by older adults’ family members and social communities were important for increasing vaccination rates [18]. Therefore, the variable of social connection in which the marital status played an important role [59] was a factor that cannot be ignored in future studies on vaccination.

### Practical Applications

This study provided evidence for practical applications in reducing vaccine hesitancy among older adults. Our findings suggested that increasing the vaccination willingness of older adults was strongly dependent on increasing confidence, both in the vaccine and in health care workers. Future interventions should focus on increasing vaccine recommendations by health care workers, especially those in primary care, on which older adults rely heavily [60]. Moreover, the low-uptake vaccines such as the influenza, pneumonia, and herpes zoster should be promoted by the government as much as the COVID-19 vaccine, given that they also correspond to extremely damaging diseases for older adults.

Further, since some older adults do not use social media and are distrustful of pharmaceutical interventions, future interventions among older adults should focus on instilling information about the safety, importance, and necessity of vaccines. The convenience dimension was a variable that required special attention in the older adult population compared with other groups. Increasing the convenience of vaccination, such as opening convenient vaccination sites, increasing reimbursement rate by health insurance, and increasing transportation convenience measures, can also reduce vaccine hesitancy among older adults [2].

Finally, awareness of the severity of vaccine-preventable diseases and infection risk should be increased, especially among older adults with low SES. Complacency was a substitute factor for SES, and our findings provided evidence that reducing complacency could eliminate the large impact of SES—a variable that was essentially immutable in older populations—on vaccination intentions.

### Limitations

Selection bias is one possible limitation. Although all older adults were required to participate in this free annual physical examination where we recruited participants, it was not mandatory. Therefore, the individuals who participated in this survey might have been more concerned about health care than their counterparts. However, we controlled for demographic factors in our analysis. In addition, the AVE of convenience for the questionnaire was slightly lower than the related correlation with complacency; however, the overall model fit was ideal. This questionnaire should be improved in future studies. All the data were self-reported, and realistic data such as vaccine efficacy and safety were not included in this study. This study was conducted in Jiangsu Province, an economically developed province in China; hence, extrapolation need to be considered with great caution, especially in less economically developed areas. In these areas, for example, the association between convenience and older adults’ vaccine hesitancy may increase positively.

### Conclusion

This study developed a model based on the 3C theory including SES and vaccination history as moderating variables to explore the drivers of vaccine hesitancy among older adults. Confidence was the more influential factor in reducing vaccine hesitancy among older adults. Our findings also highlighted that COVID-19 vaccination history, which had a positive influence on less vaccine hesitancy, weakened the role of confidence in vaccine hesitancy. The substitution relationship between SES and complacency suggested a competitive positive effect of SES and less complacency on less vaccine hesitancy. These associations could provide potential strategies that can be used to mitigate vaccine hesitancy for older adults with different reasons for SES, confidence, and complacency.

## Appendix

Multimedia Appendix 1 Supplementary materials.

## Abbreviations

AGFI: adjusted goodness-of-fit index
AVE: average variance extracted
CFI: comparative fit
GFI: goodness-of-fit index
RMSEA: the root means square error of approximation
SES: socioeconomic status
3Cs: confidence, complacency, and convenience

## Data Availability

The data sets generated during the current study are not publicly available due to their privacy but are available from the corresponding author on reasonable request.
