# Supplementary material for: Understanding Drivers of Vaccine Hesitancy During the COVID-19 Pandemic Among Older Adults in Jiangsu Province, China: Cross-sectional Survey
Source: JMIR Form Res. 2023 Feb 7;7:e39994. doi: 10.2196/39994 (PMC9907572; doi:10.2196/39994)
Supplement: Multimedia Appendix 1 [file formative_v7i1e39994_app1.docx]

**Multimedia Appendix**

**Material S1:** The Calculation of Sample Size

**Material S2:** The Original Questionnaire

**Material S3:** Consisting Variables in the Hierarchical moderator regression analysis

**Figure S1:** The inclusion and exclusion criteria of the participants

**Table S1.** The Modified Questionnaire

**Table S2.** The Reliability of Items

**Table S3.** The Validity of the Constructs

**Table S4.** The Estimate of Variances

**Table S5.** The Estimate of Residual Covariation

**Table S6.** Model Information in Hierarchical Moderator Regression Analysis (original data where missing values were not imputed)

**Table S7.** Variable Coefficients of Block 3 in Hierarchical Moderator Regression Analysis (original data where missing values were not imputed)

**Table S8.** Model Information in Hierarchical Moderator Regression Analysis (missing values were deleted)

**Table S9.** Variable Coefficients of Block 3 in Hierarchical Moderator Regression Analysis (missing values were deleted)

**Table S10.** Effects on Structural Equation Model (original data where missing values were not imputed)

**Table S11.** Effects on Structural Equation Model CC (missing values were deleted)

**Material S1: The Calculation of Sample Size**

Type I error α was set to 5%, the permissible error d was 0.05, the design effect (deff) was 2, and p was the vaccine hesitation rate, which was set to 50% (considering the maximum sample size). Considering factors such as dropping out and exclusion, the sample size was expanded by 30%, and the final sample size was 999.

**Material S2: The Original Questionnaire**

The first section of the questionnaire was demographic (age, gender, marriage statement, self-assessment of financial situation), socioeconomic status (education, occupation, medical career background, revenue), and health information (chronic disease history, self-assessment of health status, vaccination history).

The second section was the construction of the 3Cs model, which includes confidence, convenience, and complacency. All items within each construct were chosen according to MacDonald’s view [1] on the definition of the 3Cs model, as follows:

1. confidence is defined as trust in the effectiveness and safety of vaccines; the system that delivers them, including the reliability and competence of the health services and health professionals and the motivations of policy-makers who decide on the needed vaccines.
2. Complacency exists where perceived risks of vaccine-preventable diseases are low and vaccination is not deemed a necessary preventive action.
3. Convenience is a significant factor when physical availability, affordability and willingness-to-pay, geographical accessibility, ability to understand (language and health literacy) and appeal of immunization services affect uptake. The quality of the service (real and/or perceived) and the degree to which vaccination services are delivered at a time and place and in a cultural context that is convenient and comfortable also affect the decision to be vaccinated and could lead to vaccine hesitancy.

| **Dimension** | **Questions** | **Scale** |
| --- | --- | --- |
| **Confidence** | Q1. I think that vaccination is safe. | from 1 (strongly disagree) to 5 (strongly agree) |
|  | Q2. I think that vaccination is effective. |  |
|  | Q3. I think that vaccination is important. |  |
|  | Q4. I believe that the full chain of vaccine management is safe and effective. |  |
|  | Q5. I trust doctors and nurses | from 1 (strongly distrust) to 5 (strongly trust) |
|  | Q6. I trust hospitals and community vaccination clinics |  |
|  | Q7. I trust vaccine manufacturers |  |
|  | Q8. I trust the vaccine information provided by the government |  |
| **Complacency** | Q9. If I do not get vaccinated, I won't get the disease. | from 1 (strongly disagree) to 5 (strongly agree) |
|  | Q10. Natural immunity is better than that produced by vaccination. |  |
|  | Q11. The probability of getting diseases is low, so I do not need to get vaccinated. |  |
|  | Q12. Even if I get infected with a disease I can resist it, so I don't need to be vaccinated. |  |
| **Convenience** | Q13. The poor quality of service at the vaccine clinic would make me not want to go for vaccination. | from 1 (strongly disagree) to 5 (strongly agree) |
|  | Q14. It was easy and took me a short time to get the vaccination. |  |
|  | Q15. I can get the vaccine that I want. |  |
|  | Q16. I can afford the vaccine. |  |

The third section was the dimension of vaccine hesitancy.

| **Dimension** | **Questions** | **Scale** |
| --- | --- | --- |
| **Vaccine hesitancy** | Q17. How likely would you go for a COVID-19 vaccine? | from 1 (completely impossible) to 5 (completely possible) |
|  | Q18. Would you get an inﬂuenza shot this year? |  |
|  | Q19. If you could now get an influenza vaccine at your own expense, what is your choice? | from 1 (refusing to get it) to 5 (getting it right away) [2 (trying to postpone it), 3 (not sure), 4 (getting it when I have time) |

Each item in the second and third sections was assessed using a 5-point Likert, ranging from 1 (strongly disagree, completely impossible, etc.) to 5 (strongly agree, completely possible, etc.).

Reference:

[1] MacDonald NE; SAGE Working Group on Vaccine Hesitancy. Vaccine hesitancy: Definition, scope and determinants. Vaccine. 2015;33(34):4161-4164. doi:10.1016/j.vaccine.2015.04.036

**Material S3: Consisting Variables in the Hierarchical moderator regression analysis**

Control variables were entered as block 1 (age, gender, marriage statement, chronic disease history, self-assessment of health status, self-assessment of economic status), followed by the standardized values of the main effects (confidence, convenience, complacency, socioeconomic status, and vaccination history) in block 2. Finally, the moderating effects between socioeconomic status and vaccination history with confidence, convenience, and complacency were used as block 3.

Regression equation is as follows:

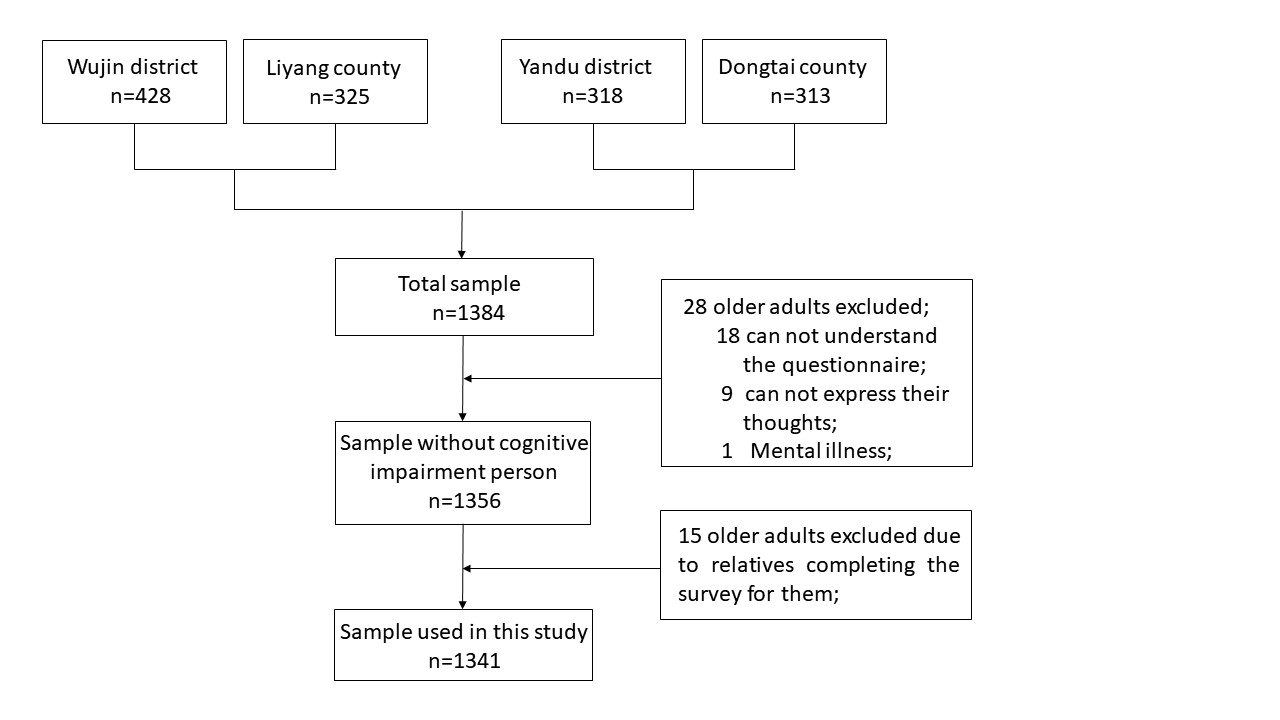


**Figure S1: The inclusion and exclusion criteria of the participants**

**Table S1. The Modified Questionnaire**

| **Dimension** | **Questions** | **Scale** |
| --- | --- | --- |
| **Confidence** | ConfidenceⅠ | from 1 (strongly disagree) to 5 (strongly agree) |
|  | Q1. I think that vaccination is safe. |  |
|  | Q2. I think that vaccination is effective. |  |
|  | Q3. I think that vaccination is important. |  |
|  | ConfidenceⅡ | from 1 (strongly distrust) to 5 (strongly trust) |
|  | Q4. I trust doctors and nurses |  |
|  | Q5. I trust hospitals and community vaccination clinics |  |
|  | Q6. I trust vaccine manufacturers |  |
|  | Q7. I trust the vaccine information provided by the government |  |
| **Complacency** | Q8. Natural immunity is better than that produced by vaccination. | from 1 (strongly disagree) to 5 (strongly agree) |
|  | Q9. The probability of getting diseases is low, so I do not need to get vaccinated. |  |
|  | Q10. Even if I get infected with a disease I can resist it, so I don't need to be vaccinated. |  |
| **Convenience** | Q11. The poor quality of service at the vaccine clinic would make me not want to go for vaccination. | from 1 (strongly disagree) to 5 (strongly agree) |
|  | Q12. It was easy and took me a short time to get the vaccination. |  |
|  | Q13. I can get the vaccine that I want. |  |
|  | Q14. I can afford the vaccine. |  |
| **Vaccine hesitancy** | Q17. How likely would you go for a COVID-19 vaccine? | from 1 (completely impossible) to 5 (completely possible) |
|  | Q18. Would you get an inﬂuenza shot this year? |  |
|  | Q19. If you could now get an influenza vaccine at your own expense, what is your choice? | from 1 (refusing to get it) to 5 (getting it right away) [2 (trying to postpone it), 3 (not sure), 4 (getting it when I have time) |

**Table S2. The Reliability of Items**

| **Dimension** | **Parameter significance estimation** | | | | **Factor loading** | **SMC** ^d^ | **CR** ^e^ | **AVE** ^f^ |
| --- | --- | --- | --- | --- | --- | --- | --- | --- |
|  | **Unstd.**^a^ | **S.E.** ^b^ | **t-value** | **p-value** | **Std.** ^c^ |  |  |  |
| **Confidence**  ConfidenceⅠ^g^ | | | | | | | | |
| Q1 | 1.000 | | | | 0.819 | 0.671 | 0.786 | 0.556 |
| Q2 | 1.028 | 0.048 | 21.285 | <.001 | 0.805 | 0.648 |  |  |
| Q3 | 0.717 | 0.037 | 19.215 | <.001 | 0.591 | 0.349 |  |  |
| ConfidenceⅡ^h^ | | | | | | | | |
| Q4 | 1.000 |  |  |  | 0.903 | 0.815 | 0.814 | 0.542 |
| Q5 | 1.048 | 0.029 | 36.021 | <.001 | 0.914 | 0.835 |  |  |
| Q6 | 0.762 | 0.039 | 19.421 | <.001 | 0.509 | 0.259 |  |  |
| Q7 | 0.515 | 0.026 | 19.426 | <.001 | 0.509 | 0.259 |  |  |
| **Complacency** | | | | | | | | |
| Q8 | 1.000 | | | | 0.549 | 0.301 | 0.818 | 0.609 |
| Q9 | 1.518 | 0.076 | 19.856 | <.001 | 0.859 | 0.738 |  |  |
| Q10 | 1.602 | 0.082 | 19.539 | <.001 | 0.887 | 0.787 |  |  |
| **Convenience** | | | | | | | | |
| Q11 | 1.000 | | | | 0.532 | 0.283 | 0.740 | 0.424 |
| Q12 | 1.486 | 0.091 | 16.294 | <.001 | 0.772 | 0.596 |  |  |
| Q13 | 1.147 | 0.071 | 16.260 | <.001 | 0.737 | 0.543 |  |  |
| Q14 | 0.921 | 0.067 | 13.693 |  | 0.522 | 0.272 |  |  |
| **Vaccine hesitancy** | | | | | | | | |
| Q15 | 1.000 | | | | 0.901 | 0.812 | 0.732 | 0.497 |
| Q16 | 0.436 | 0.036 | 12.244 | <.001 | 0.430 | 0.185 |  |  |
| Q17 | 0.643 | 0.044 | 14.629 | <.001 | 0.721 | 0.493 |  |  |

1. Unstd. is the abbreviation of unstandardized estimate
2. S.E. is the abbreviation of standard error
3. Std. is the abbreviation of standardized estimate
4. SMC is the abbreviation of squared multiple correlations
5. CR is the abbreviation of composite reliability
6. AVE is the abbreviation of average variances extracted
7. ConfidenceⅠmeans the confidence in vaccines
8. ConfidenceⅡmeans the confidence in healthcare workers and vaccine manufacturers

**Table S3. The Validity of the Constructs**

| **Dimension** | **AVE** | **hesitancy** | **convenience** | **complacency** | **ConfidenceⅡ**^a^ | **ConfidenceⅠ**^b^ |
| --- | --- | --- | --- | --- | --- | --- |
| **Hesitancy** | 0.497 | 0.705 |  |  |  |  |
| **Convenience** | 0.424 | 0.303 | 0.651 |  |  |  |
| **Complacency** | 0.609 | 0.182 | 0.680 | 0.780 |  |  |
| **ConfidenceⅡ** | 0.542 | 0.233 | 0.279 | 0.201 | 0.736 |  |
| **ConfidenceⅠ** | 0.556 | 0.320 | 0.337 | 0.204 | 0.448 | 0.746 |

1. ConfidenceⅡmeans the confidence in healthcare workers and vaccine manufacturers
2. ConfidenceⅠmeans the confidence in vaccines

**Table S4. The Estimate of Variances**

| **Dimension** | **Item** | **Variances** | |
| --- | --- | --- | --- |
|  |  | **Estimate** | **p-value** |
| Confidence | Q1 | 0.201 | < 0.001 |
|  | Q2 | 0.153 | <0.001 |
|  | Q3 | 0.309 | <0.001 |
|  | Q4 | 0.071 | <0.001 |
|  | Q5 | 0.078 | <0.001 |
|  | Q6 | 0.533 | <0.001 |
|  | Q7 | 0.244 | <0.001 |
| Complacency | Q8 | 0.776 | <0.001 |
|  | Q9 | 0.305 | <0.001 |
|  | Q10 | 0.222 | <0.001 |
| Convenience | Q11 | 0.898 | <0.001 |
|  | Q12 | 0.636 | <0.001 |
|  | Q13 | 0.435 | <0.001 |
|  | Q14 | 0.789 | <0.001 |
| Hesitancy | Q15 | 0.657 | <0.001 |
|  | Q16 | 1.534 | <0.001 |
|  | Q17 | 0.570 | <0.001 |

**Table S5. The Estimate of Residual Covariation**

| **Items** | | | **M.I.** | **Par Change** | **Items** | | | **M.I.** | **Par Change** |
| --- | --- | --- | --- | --- | --- | --- | --- | --- | --- |
| e_hesitancy | <--> | confidence | 27.497 | 0.079 | e_Q7 | <--> | e_Q15 | 7.193 | -0.034 |
| e_hesitancy | <--> | complacency | 15.313 | -0.054 | e_Q7 | <--> | e_Q13 | 10.313 | -0.033 |
| e_confidence_Q1 | <--> | e_hesitancy | 15.869 | 0.060 | e_Q7 | <--> | e_Q8 | 6.493 | -0.032 |
| e_Q17 | <--> | confidence | 9.737 | 0.054 | e_Q6 | <--> | confidence | 9.552 | 0.036 |
| e_Q17 | <--> | complacency | 5.500 | -0.037 | e_Q6 | <--> | convenience | 13.065 | 0.044 |
| e_Q17 | <--> | e_confidence_Q1 | 9.260 | 0.053 | e_Q6 | <--> | e_hesitancy | 8.269 | 0.056 |
| e_Q16 | <--> | confidence | 21.284 | 0.091 | e_Q6 | <--> | e_Q20 | 12.555 | -0.039 |
| e_Q16 | <--> | e_confidence_Q1 | 13.116 | 0.072 | e_Q6 | <--> | e_confidence_Q1 | 29.174 | 0.063 |
| e_Q14 | <--> | confidence | 12.585 | 0.052 | e_Q6 | <--> | e_Q16 | 5.583 | 0.061 |
| e_Q14 | <--> | convenience | 4.852 | -0.033 | e_Q6 | <--> | e_Q10 | 7.231 | 0.035 |
| e_Q14 | <--> | e_hesitancy | 74.787 | 0.212 | e_Q6 | <--> | e_Q9 | 7.833 | -0.037 |
| e_Q14 | <--> | e_Q20 | 5.003 | 0.031 | e_Q6 | <--> | e_Q7 | 9.308 | 0.031 |
| e_Q14 | <--> | e_Q17 | 26.622 | 0.146 | e_Q5 | <--> | confidence | 17.253 | -0.023 |
| e_Q14 | <--> | e_Q16 | 11.832 | -0.111 | e_Q5 | <--> | convenience | 8.703 | 0.018 |
| e_Q14 | <--> | e_Q15 | 14.239 | 0.088 | e_Q5 | <--> | e_confidence_Q1 | 29.498 | -0.031 |
| e_Q13 | <--> | complacency | 6.068 | -0.026 | e_Q5 | <--> | e_Q14 | 10.784 | 0.030 |
| e_Q13 | <--> | e_Q16 | 4.585 | 0.055 | e_Q5 | <--> | e_Q13 | 9.169 | 0.022 |
| e_Q12 | <--> | e_hesitancy | 16.160 | -0.096 | e_Q5 | <--> | e_Q12 | 4.175 | -0.018 |
| e_Q12 | <--> | e_Q20 | 6.703 | -0.035 | e_Q4 | <--> | convenience | 8.867 | -0.017 |
| e_Q12 | <--> | e_Q17 | 17.541 | -0.116 | e_Q4 | <--> | e_Q14 | 8.997 | -0.027 |
| e_Q12 | <--> | e_Q16 | 5.976 | 0.077 | e_Q4 | <--> | e_Q13 | 4.115 | -0.014 |
| e_Q12 | <--> | e_Q14 | 17.715 | -0.096 | e_Q4 | <--> | e_Q10 | 9.436 | -0.019 |
| e_Q12 | <--> | e_Q13 | 17.521 | 0.074 | e_Q4 | <--> | e_Q9 | 10.574 | 0.020 |
| e_Q11 | <--> | convenience | 10.520 | -0.052 | e_Q4 | <--> | e_Q8 | 5.596 | 0.020 |
| e_Q11 | <--> | complacency | 29.308 | 0.076 | e_Q4 | <--> | e_Q6 | 7.781 | -0.019 |
| e_Q11 | <--> | e_hesitancy | 12.496 | -0.092 | e_Q3 | <--> | convenience | 17.688 | -0.040 |
| e_Q11 | <--> | e_Q15 | 10.329 | -0.080 | e_Q3 | <--> | complacency | 22.112 | 0.039 |
| e_Q11 | <--> | e_Q13 | 21.178 | -0.092 | e_Q4 | <--> | e_Q8 | 5.596 | 0.020 |
| e_Q11 | <--> | e_Q12 | 4.140 | 0.050 | e_Q4 | <--> | e_Q6 | 7.781 | -0.019 |
| e_Q10 | <--> | e_Q11 | 6.032 | 0.043 | e_Q3 | <--> | convenience | 17.688 | -0.040 |
| e_Q9 | <--> | convenience | 4.461 | -0.023 | e_Q3 | <--> | complacency | 22.112 | 0.039 |
| e_Q9 | <--> | e_hesitancy | 5.788 | -0.041 | e_Q3 | <--> | e_hesitancy | 5.419 | 0.036 |
| e_Q9 | <--> | e_Q17 | 4.679 | -0.043 | e_Q3 | <--> | e_Q20 | 10.615 | 0.028 |
| e_Q8 | <--> | convenience | 31.592 | 0.084 | e_Q3 | <--> | e_Q16 | 24.180 | 0.100 |
| e_Q8 | <--> | complacency | 12.007 | -0.044 | e_Q3 | <--> | e_Q14 | 4.308 | 0.031 |
| e_Q8 | <--> | e_hesitancy | 8.543 | -0.070 | e_Q3 | <--> | e_Q12 | 24.630 | -0.072 |
| e_Q8 | <--> | e_Q15 | 4.617 | -0.049 | e_Q3 | <--> | e_Q10 | 17.094 | 0.043 |
| e_Q8 | <--> | e_Q12 | 12.428 | 0.079 | e_Q3 | <--> | e_Q7 | 24.280 | 0.040 |
| e_Q8 | <--> | e_Q11 | 10.396 | 0.079 | e_Q2 | <--> | complacency | 4.377 | -0.015 |
| e_Q8 | <--> | e_Q10 | 4.428 | -0.033 | e_Q2 | <--> | e_Q16 | 7.803 | 0.048 |
| e_Q7 | <--> | confidence | 31.588 | 0.044 | e_Q2 | <--> | e_Q15 | 5.985 | -0.031 |
| e_Q7 | <--> | convenience | 10.113 | -0.026 | e_Q2 | <--> | e_Q7 | 11.927 | 0.024 |
| e_Q7 | <--> | e_hesitancy | 7.564 | 0.036 | e_Q2 | <--> | e_Q6 | 8.717 | 0.030 |
| e_Q7 | <--> | e_Q20 | 5.729 | -0.018 | e_Q2 | <--> | e_Q5 | 7.511 | -0.013 |
| e_Q7 | <--> | e_confidence_Q1 | 50.560 | 0.056 | e_Q1 | <--> | e_Q20 | 10.028 | -0.025 |
| e_Q7 | <--> | e_Q17 | 8.556 | 0.045 | e_Q1 | <--> | e_Q16 | 8.170 | -0.053 |
| e_Q7 | <--> | e_Q16 | 13.373 | 0.064 |  |  |  |  |  |

**Table S6. Model Information in Hierarchical Moderator Regression Analysis (original data where missing values were not imputed)**

| **Model** | **Statistics estimate** | | | **Statistics change** | | |
| --- | --- | --- | --- | --- | --- | --- |
|  | **R^2^** | **adjusted R^2^** | **S.E.** ^a^ | ∆**R^2^** | **F-value** | **P-value** |
| **Block 1**^b^ | 0.054 | 0.054 | 0.975 | 0.054 | 5.950 | *P* <.001 |
| **Block 2**^c^ | 0.215 | 0.205 | 0.890 | 0.161 | 50.743 | *P* <.001 |
| **Block 3**^d^ | 0.234 | 0.219 | 0.882 | 0.018 | 4.856 | *P* <.001 |

1. S.E. is the abbreviation of standard error
2. The variables in block 1 were age, marital status, gender, self-assessment of the financial situation, self-assessment of health status, and chronic diseases.
3. The variables in block 2 was age, marital status, gender, self-assessment of the financial situation, self-assessment of health status, chronic diseases, standardized of confidence, convenience, and complacency, and standardized of socioeconomic status and vaccine history.
4. The variables in block 3 was age, marital status, gender, self-assessment of the financial situation, self-assessment of health status, chronic diseases, standardized of confidence, convenience, and complacency, standardized of socioeconomic status and vaccine history, socioeconomic status*convenience, socioeconomic status*confidence, socioeconomic status*complacency, vaccine history* convenience, vaccine history*confidence, vaccine history*complacency.

**Table S7. Variable Coefficients of Block 3 in Hierarchical Moderator Regression Analysis (original data where missing values were not imputed)**

| **Variables** | **Unstd.** ^a^ | | **Std.** ^c^ | **t-value** | **p-value** |
| --- | --- | --- | --- | --- | --- |
|  | **b** | **S.E.** ^b^ |  |  |  |
| **Constant** | -0.160 | 0.585 |  | -0.274 | *P* = .78 |
| **Age** | -0.009 | 0.005 | -0.051 | -1.894 | *P* = .06 |
| **Gender** ^d^ | -0.021 | 0.052 | -0.010 | -0.400 | *P* = .69 |
| **Marital status** ^e^ | -0.181 | 0.067 | -0.071 | -2.703 | *P* = .007 |
| **Chronic diseases** ^f^ | 0.037 | 0.053 | 0.019 | 0.705 | *P* = .48 |
| **Self-assessment of the financial situation (“very generous” as ref)** | | | | | |
| generous | 0.194 | 0.295 | 0.057 | 0.657 | *P* = .51 |
| roughly adequate | 0.115 | 0.289 | 0.057 | 0.396 | *P* = .69 |
| tough | 0.046 | 0.292 | 0.022 | 0.158 | *P* = .87 |
| very tough | 0.079 | 0.317 | 0.016 | 0.250 | *P* = .80 |
| **Self-assessment of health status (“very poor” as ref)** | | | | | |
| poor | 0.749 | 0.371 | 0.288 | 2.021 | *P* = .04 |
| general | 0.784 | 0.369 | 0.369 | 2.122 | *P* = .03 |
| well | 0.681 | 0.369 | 0.340 | 1.846 | *P* = .07 |
| very well | 0.443 | 0.389 | 0.087 | 1.140 | *P* = .26 |
| **Socioeconomic status** | 0.135 | 0.033 | 0.133 | 4.052 | *P* < .001 |
| **Vaccination history** | 0.082 | 0.027 | 0.083 | 2.986 | *P* = .003 |
| **Confidence** | 0.227 | 0.028 | 0.228 | 8.176 | *P* <.001 |
| **Complacency** | -0.004 | 0.031 | -0.004 | -0.132 | *P* = .90 |
| **Convenience** | 0.306 | 0.034 | 0.310 | 8.930 | *P* <.001 |
| **Socioeconomic status* Confidence** | 0.094 | 0.028 | 0.091 | 3.376 | *P* = .001 |
| **Socioeconomic status* Convenience** | -0.067 | 0.031 | -0.071 | -2.177 | *P* = .03 |
| **Socioeconomic status* Complacency** | -0.068 | 0.030 | -0.068 | -2.218 | *P* = .03 |
| **Vaccination history * Confidence** | -0.040 | 0.028 | -0.040 | -1.403 | *P* = .16 |
| **Vaccination history * Convenience** | 0.053 | 0.031 | 0.057 | 1.716 | *P* = .09 |
| **Vaccination history * Complacency** | -0.028 | 0.032 | -0.028 | -0.875 | *P* = .38 |

1. Unstd. is the abbreviation of unstandardized estimate
2. S.E. is the abbreviation of standard error
3. Std. is the abbreviation of standardized estimate
4. Gender is a binary variable, and use “male” as reference
5. Marital status is changed into a binary variable, of which unmarried, divorced, and widowed are combined into “single”.
6. Chronic disease is a binary variable, and use “yes” as reference

**Table S8. Model Information in Hierarchical Moderator Regression Analysis (missing values were deleted)**

| **Model** | **Statistics estimate** | | | **Statistics change** | | |
| --- | --- | --- | --- | --- | --- | --- |
|  | **R^2^** | **adjusted R^2^** | **S.E.** ^a^ | ∆**R^2^** | **F-value** | **P-value** |
| **Block 1**^b^ | 0.055 | 0.045 | 0.976 | 0.055 | 5.496 | *P* <.001 |
| **Block 2**^c^ | 0.218 | 0.206 | 0.890 | 0.163 | 47.533 | *P* <.001 |
| **Block 3**^d^ | 0.237 | 0.221 | 0.882 | 0.019 | 4.631 | *P* <.001 |

1. S.E. is the abbreviation of standard error
2. The variables in block 1 were age, marital status, gender, self-assessment of the financial situation, self-assessment of health status, and chronic diseases.
3. The variables in block 2 was age, marital status, gender, self-assessment of the financial situation, self-assessment of health status, chronic diseases, standardized of confidence, convenience, and complacency, and standardized of socioeconomic status and vaccine history.
4. The variables in block 3 was age, marital status, gender, self-assessment of the financial situation, self-assessment of health status, chronic diseases, standardized of confidence, convenience, and complacency, standardized of socioeconomic status and vaccine history, socioeconomic status*convenience, socioeconomic status*confidence, socioeconomic status*complacency, vaccine history* convenience, vaccine history*confidence, vaccine history*complacency.

**Table S9. Variable Coefficients of Block 3 in Hierarchical Moderator Regression Analysis (missing values were deleted)**

| **Variables** | **Unstd.** ^a^ | | **Std.** ^c^ | **t-value** | **p-value** |
| --- | --- | --- | --- | --- | --- |
|  | **b** | **S.E.** ^b^ |  |  |  |
| **Constant** | -0.161 | 0.600 |  | -0.268 | *P* = .79 |
| **Age** | -0.010 | 0.005 | -0.056 | -1.980 | *P* = .05 |
| **Gender** ^d^ | -0.029 | 0.055 | -0.014 | -0.533 | *P* = .59 |
| **Marital status** ^e^ | -0.176 | 0.070 | -0.069 | -2.518 | *P* = .01 |
| **Chronic diseases** ^f^ | 0.023 | 0.056 | 0.011 | 0.416 | *P* = .68 |
| **Self-assessment of the financial situation (“very generous” as ref)** | | | | | |
| generous | 0.303 | 0.312 | 0.087 | 0.972 | *P* = .33 |
| roughly adequate | 0.204 | 0.305 | 0.102 | 0.669 | *P* = .50 |
| tough | 0.143 | 0.308 | 0.066 | 0.463 | *P* = .64 |
| very tough | 0.161 | 0.335 | 0.031 | 0.480 | *P* = .63 |
| **Self-assessment of health status (“very poor” as ref)** | | | | | |
| poor | 0.754 | 0.372 | 0.291 | 2.029 | *P* = .04 |
| general | 0.790 | 0.371 | 0.372 | 2.129 | *P* = .03 |
| well | 0.675 | 0.370 | 0.336 | 1.822 | *P* = .07 |
| very well | 0.454 | 0.393 | 0.087 | 1.155 | *P* = .25 |
| **Socioeconomic status** | 0.130 | 0.035 | 0.129 | 3.757 | *P* < .001 |
| **Vaccination history** | 0.084 | 0.029 | 0.084 | 2.912 | *P* = .004 |
| **Confidence** | 0.233 | 0.029 | 0.230 | 7.933 | *P* <.001 |
| **Complacency** | -0.004 | 0.033 | -0.004 | -0.115 | *P* = .91 |
| **Convenience** | 0.306 | 0.036 | 0.312 | 8.585 | *P* <.001 |
| **Socioeconomic status* Confidence** | 0.096 | 0.030 | 0.090 | 3.225 | *P* = .001 |
| **Socioeconomic status* Convenience** | -0.063 | 0.032 | -0.068 | -1.976 | *P* = .05 |
| **Socioeconomic status* Complacency** | -0.074 | 0.032 | -0.075 | -2.313 | *P* = .02 |
| **Vaccination history * Confidence** | -0.050 | 0.031 | -0.048 | -1.619 | *P* = .11 |
| **Vaccination history * Convenience** | 0.047 | 0.032 | 0.051 | 1.465 | *P* = .14 |
| **Vaccination history * Complacency** | -0.021 | 0.034 | -0.022 | -0.629 | *P* = .53 |

1. Unstd. is the abbreviation of unstandardized estimate
2. S.E. is the abbreviation of standard error
3. Std. is the abbreviation of standardized estimate
4. Gender is a binary variable, and use “male” as reference
5. Marital status is changed into a binary variable, of which unmarried, divorced, and widowed are combined into “single”
6. Chronic disease is a binary variable, and use “yes” as reference

**Table S10. Effects on Structural Equation Model (original data where missing values were not imputed)**

| **Direct Effects** | **Point estimate（std.** ^a^**）** | **S.E.** ^b^ | **Point estimate（unstd.** ^c^**）** | **p-value** |
| --- | --- | --- | --- | --- |
| **Model 1** |  | | |  |
| Confidence--Vaccine Hesitancy | 0.453 | 0.109 | 0.970 | *P* < .001 |
| **Model 2** |  |  |  |  |
| Complacency--Vaccine Hesitancy | 0.203 | 0.050 | 0.301 | *P* < .001 |
| **Model 3** |  |  |  |  |
| Convenience--Vaccine Hesitancy | 0.328 | 0.054 | 0.463 | *P* < .001 |

1. Std. is the abbreviation of standardized estimate
2. Unstd. is the abbreviation of unstandardized estimate
3. S.E. is the abbreviation of standard error

**Table S11. Effects on Structural Equation Model CC (missing values were deleted)**

| **Direct Effects** | **Point estimate（std.** ^a^**）** | **S.E.** ^b^ | **Point estimate（unstd.** ^c^**）** | | **Bootstrapping (5000 times)** | | | | |
| --- | --- | --- | --- | --- | --- | --- | --- | --- | --- |
|  |  |  |  |  | **Percentile 95%CI** ^d^ | | **Bias-corrected percentile 95%CI** | | **p-value** |
|  |  |  |  |  | **Lower** | **Upper** | **Lower** | **Upper** |  |
| **Model 1** |  | | |  | | | | | |
| Confidence--Vaccine Hesitancy | 0.462 | 0.041 | 1.001 | | 0.382 | 0.540 | 0.387 | 0.542 | *P* = .002 |
| **Model 2** |  |  |  | |  |  |  |  |  |
| Complacency--Vaccine Hesitancy | 0.206 | 0.035 | 0.306 | | 0.139 | 0.276 | 0.136 | 0.275 | *P* = .002 |
| **Model 3** |  |  |  | |  |  |  |  |  |
| Convenience--Vaccine Hesitancy | 0.325 | 0.039 | 0.476 | | 0.250 | 0.403 | 0.252 | 0.406 | *P* = .002 |
| **Full model** ^e^ | 0.117 | 0.025 | 0.224 | | 0.081 | 0.166 | 0.083 | 0.168 | *P* = .002 |

1. Std. is the abbreviation of standardized estimate
2. Unstd. is the abbreviation of unstandardized estimate
3. S.E. is the abbreviation of standard error
4. CI is the abbreviation of the confidence interval
5. the effects of confidence, convenience, and complacency were split equally.
